# Mechanically Strengthened Graphene Oxide: Covalent Organic Framework Membranes for Monovalent/Divalent Cation Selectivity via Electrodialysis

**DOI:** 10.1021/acsnano.5c17289

**Published:** 2026-01-21

**Authors:** Yuren Feng, Xiaoyin Tian, Yifan Zhu, Qiyi Fang, Rodolfo Cantu, Bongki Shin, Xiaochuan Huang, Xintong Weng, Xiang Zhang, Yunhao Zhang, Doug Steinbach, Qing Ai, Tsai-Hsuan Chen, Yimo Han, Pulickel M. Ajayan, Qilin Li, Jun Lou

**Affiliations:** † NSF Nanosystems Engineering Research Center Nanotechnology-Enabled Water Treatment, 3990 Rice University, 6100 Main Street, Houston, Texas 77005, United States; ‡ Department of Civil and Environmental Engineering, Rice University, 6100 Main Street, Houston, Texas 77005, United States; § Department of Materials Science and NanoEngineering, Rice University, 6100 Main Street, Houston, Texas 77005, United States; ∥ Graduate Institute of Environmental Engineering, 33561 National Taiwan University, No. 1, Sec. 4. Roosevelt Rd., Taipei 10617, Taiwan; ⊥ Department of Chemical & Biomolecular Engineering, Rice University, 6100 Main Street, Houston, Texas 77005, United States; # Rice Advanced Materials Institute, Rice University, 6100 Main Street, Houston, Texas 77005, United States; ¶ Rice WaTER Institute, Rice University, 6100 Main Street, Houston, Texas 77005, United States; ∇ Department of Chemistry, Rice University, 6100 Main Street, Houston, Texas 77005, United States

**Keywords:** covalent organic framework, ion separation, membrane, mechanical robustness, electrodialysis, selectivity

## Abstract

Electrodialysis is an energy efficient method for water desalination and resource recovery; however, the lack of ion selectivity in conventional ion-exchange membranes limits its performance, particularly in separating monovalent and divalent cations. In this study, we present the design and fabrication of mechanically reinforced graphene oxide (GO)–covalent organic framework (COF) composite membranes with variable selectivity for monovalent over divalent cations, enhancing the performance of electrodialysis processes. The composite membranes, created by stacking GO and sulfonate-functionalized COF nanosheets, exhibit a nacre-inspired “brick-and-mortar” structure that imparts mechanical robustness and high ion selectivity. By adjusting the GO-to- COF ratio, we achieved varying Na^+^/Ca^2+^ and Li^+^/Ca^2+^ selectivity ratios, reaching up to 15.34 and 6.99, respectively, without compromising the charge efficiency (greater than 75%). The membranes have also shown good stability in synthetic hypersaline brine. These results demonstrate the potential of GO/COF membranes for high-performance, energy-efficient ion separation in electrodialysis, offering a promising solution for desalination, wastewater treatment, and resource recovery.

## Graphical abstract

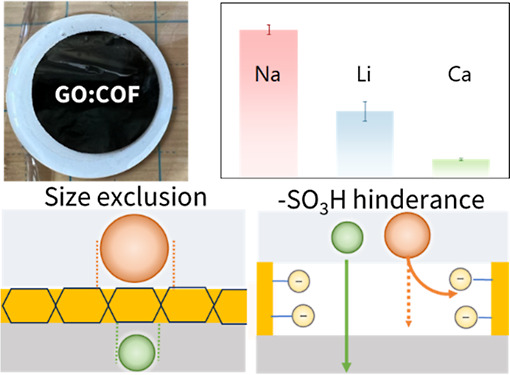

## Introduction

Ion selectivity is a crucial factor in optimizing water treatment and resource recovery processes, directly affecting system efficiency and product quality. Electrodialysis (ED), a mature technology in use for over five decades, efficiently reduces salinity in feedwater by driving ions through ion exchange membranes (IEMs) under an electric field.^1,2^ Operating under ambient conditions, ED is energy-efficient for treating saline water with a total dissolved solids (TDS) of up to 10,000 mg/L and achieves water recovery rates exceeding 85% without extensive pretreatment.^3^ Its less extensive pretreatment, higher energy efficiency, and simpler operation further distinguish it from reverse osmosis (RO), offering higher water and salt recovery with lower chemical and energy demands.^3^ However, the limited and fixed selectivity of conventional IEMs between ions with similar charges restricts ED’s adaptability to diverse water sources and end-use requirements.^4^ Due to a greater Coulomb force generated by a higher charge, divalent ions preferentially permeate through current IEMs. While this can lead to water being depleted of essential minerals like calcium (Ca^2+^) and magnesium (Mg^2+^), different applications require different levels of mineral retention—drinking water needs moderate hardness (>40 mg/L as CaCO_3_) to prevent pipeline corrosion, agricultural irrigation requires adequate Ca^2+^ and Mg^2+^ for soil health, while industrial applications may need minimal mineral content.^5–7^ Similarly, in resource recovery, the optimal balance between monovalent and divalent ion separation varies with feedwater composition and target product specifications.^8–11^ Therefore, IEMs with selectivity for monovalent ions versus divalent ions are essential to optimizing ED performance across diverse desalination and wastewater treatment applications.

Efforts to enhance ion selectivity have explored size exclusion,^12,13^ charge exclusion,^14–16^ and affinity exclusion^17,18^ mechanisms. While these approaches improve selectivity, they typically produce membranes with fixed selectivity that cannot adapt to varying feedwater compositions or application requirements. Moreover, challenges remain, including reduced ion permeability, higher energy consumption, and membrane structural defects.^19^ For instance, positively charged coatings enhance monovalent selectivity, but the degree of selectivity cannot be adjusted postfabrication, and selectivity often decreases with increasing TDS due to charge screening. Similarly, nanofiltration membranes demonstrate Na^+^/Mg^2+^ selectivity but lack the conductivity required for ED and the ability to modulate selectivity based on operational needs. Developing advanced materials to function as IEMs with monovalent/divalent selectivity—where selectivity can be adjusted based on feedwater quality and desired product specifications—is essential to overcome these limitations. Such membranes would enable a “fit-for-purpose” approach, optimizing water recovery while minimizing pre- and post-treatment requirements for each specific application, thereby unlocking the full potential of ED for versatile desalination and resource

Covalent organic frameworks (COFs) are highly crystalline and porous materials constructed through dynamic covalent bonds utilizing modular organic building blocks.^20–26^ One distinctive advantage of COFs lies in their ability to fine-tune the channel size within the range of 0.5–5.0 nm by strategically selecting linkers and linkages,^27–29^ providing an unprecedented level of freedom for constructing membranes designed for specific ion separation targets via size exclusion.^30^ Additionally, COFs boast an abundance of functional groups,^22,31–36^ enabling the incorporation of multiple physicochemical interactions that enhance their recognition ability toward target ions.^37–39^ All these properties make COFs highly suitable for use as membrane materials for ion separation.^30,40–47^ Specifically, for electrodialysis, Sun’s group reported COF membranes integrated with diverse oligoether chains, enabling highly electro-driven selective Li^+^ over Mg^2+^ transport.^48^ Very recently, Zhang’s group developed three-dimensional cationic COF membranes that exhibited exceptional Li^+^/Mg^2+^ selectivity of 321 in electrodialysis experiments, along with a high lithium permeation rate of 0.53 mol m^–2^ h^–1^.^49^ However, COF-based membranes often exhibit critical mechanical failures that prevent their industrial deployment in ED systems.^50^ Specifically, pristine COF membranes suffer from (i) catastrophic rupture under typical ED operating pressures (0.5–2 bar) due to weak π–π interlayer interactions resulting in tensile strengths below 5 MPa, which is insufficient for the 15–20 MPa pressure required in industrial modules;^51^ (ii) irreversible delamination during the membrane stack assembly process, where compression forces of 5–10 kg/cm^2^ cause layer separation and the creation of nonselective defects, leading to >90% loss in selectivity.^52^ These mechanical issues have limited COF-based membranes to bench-scale studies operating at <0.1 bar, far below the 1–5 bar operational range of commercial ED systems.^53^ To overcome these obstacles, constructing heterostructures that integrate covalent organic frameworks (COFs) with other two-dimensional (2D) materials, such as graphene oxide (GO),^54–58^ hexagonal boron nitride (hBN),^59–61^ and MXene,^62^ offers a promising strategy. These hybrid structures not only combine the distinct chemical and physical functionalities from each of their components^57,63^ but also provide opportunities to enhance the mechanical properties of membranes through structural reinforcement.^64^ Drawing inspiration from nacre (mother-of-pearl), such heterostructures can adopt a “brick-and-mortar” configuration, where rigid inorganic platelets act as the bricks and flexible organic layers function as the mortar.^64^ This arrangement imparts mechanical robustness, mitigating issues such as membrane brittleness and deformation under operational stresses. Therefore, the rational design of COF/2D inorganic material hybrid structures presents significant opportunities to develop membranes with high selectivity, exceptional water flux, and enhanced mechanical strength.

In this study, we report the fabrication of a mechanically robust electrodialysis membrane constructed by stacking GO and COF nanosheets, enabling monovalent selectivity over divalent cations. COF NUS-9,^65^ functionalized with sulfonate (–SO_3_H) groups, imparts selective ion transport properties.^41^ The negatively charged nature of both GO and the COF facilitates cation transport within the membrane, consequently enhancing the efficiency of the electrodialysis process. Furthermore, the assembly of “hard” GO nanosheets with “soft” COF layers results in a nacre-like structure, enabling the direct integration of the mechanically robust free-standing membrane into electrodialysis cells. Such a design distinguishes this work from previously reported GO/COF membranes^66,67^ as it not only enables cation exchange membrane (CEM) functionality tailored for monovalent ion electrodialysis but also significantly enhances the mechanical robustness of the membrane—an essential requirement for practical ED applications. The GO/COF composite membrane exhibits selective ion transport, with Na^+^/Ca^2+^ and Li^+^/Ca^2+^ selectivity ratios of 15.34 and 6.99, respectively. Moreover, the monovalent ion selectivity can increase from 7.88 to 15.34 by adjusting the relative composition of the COF and GO while retaining a >75% charge efficiency. These findings provide valuable insights into the design of high-performance ion-selective membranes for energy-efficient electrodialysis applications.

## Results and Discussion

The GO/COF membrane was prepared through vacuum filtration of mixtures of GO and NUS-9 COF nanosheets at various GO/COF mass ratios as described in the Materials and Methods section (Figure 1a,b). After fabrication, the GO/COF membrane was placed in DI water with a filter support to assess its stability in an aqueous environment. The membrane quickly detached from the support and remained intact, freely standing in the DI water for 24 h without decomposing, while the support settled at the bottom of the container (Figure 1c). The membrane was then clipped vertically in a three-chamber ED cell for the basic mechanical strength test since a suitable IEM for ED must separate chambers and solutions without cracking (Figure 1d). No visible cracks were observed in the membrane, nor did the water levels in either chamber change, after adding NaCl solution and DI water separately into the chambers adjacent to the GO/COF membrane over a 12 h period. These results indicate that the GO/COF membrane possesses sufficient mechanical strength for direct use in the ED process.

**Figure 1:**
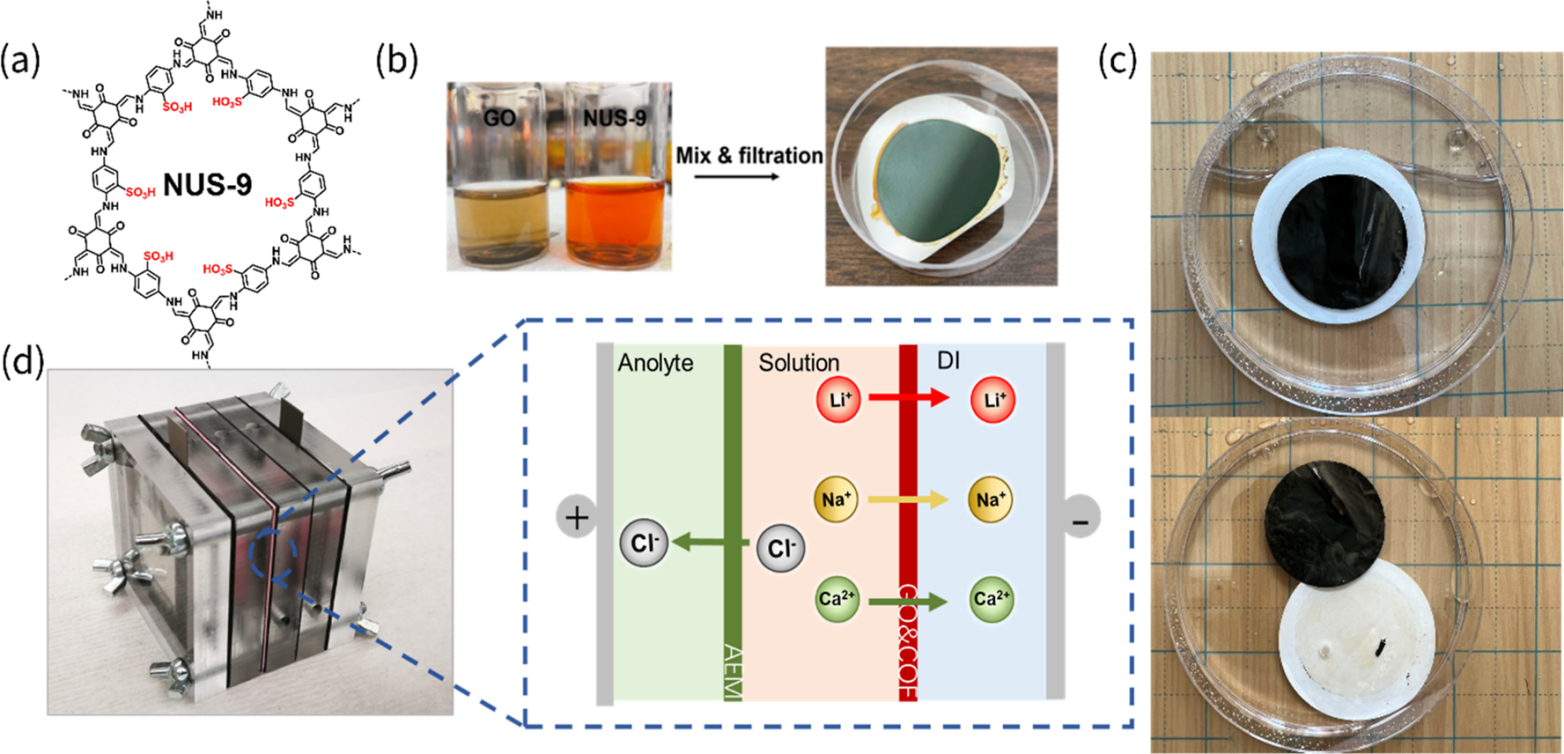
Schematic of GO/COF membranes. (a) The structure of the COF. (b) The photograph of the GO and COF solution and GO/COF membrane after filtration fabrication. (c) A visual validation of the free-standing feature of the GO/COF membrane after water assisted peeling. (d) The ED cell picture shows the schematic of the ED process with the GO/COF membrane installed.

Top-view scanning electron microscopy (SEM) images of the pure GO membrane (Figure 2a1) revealed a rough surface with prominent wrinkled corrugations, likely resulting from GO aggregation and swelling.^54^ As the COF composition increased (Figure 2a2–a4), the surface wrinkles of the hybrid membrane became less pronounced. Cross-sectional SEM imaging confirmed a layer-by-layer stacking structure in all membranes (Figure 2b1–b4). Notably, the cross-sectional SEM image of the GO/COF hybrid membranes exhibited a more compact and uniformly aligned layer configuration (Figure 2b2,b3) compared to the less ordered and loosely stacked layers observed in the pure GO membrane (Figure 2b1). Atomic force microscopy (AFM) was employed to further examine the surface morphology of the membranes (Figure 2c1–c4). The AFM images reveal that pure GO (Figure 2c1), the GO/COF membrane with low COF content (GO/COF = 20:1, Figure 2c2), and pure COF (Figure 2c4) membranes exhibit relatively rough surfaces formed by aggregated nanosheets. The AFM image of the GO/COF hybrid membrane with a higher COF content (GO/COF = 20:3) shows a relatively smooth surface with minimal particle aggregation (Figure 2c3).

**Figure 2:**
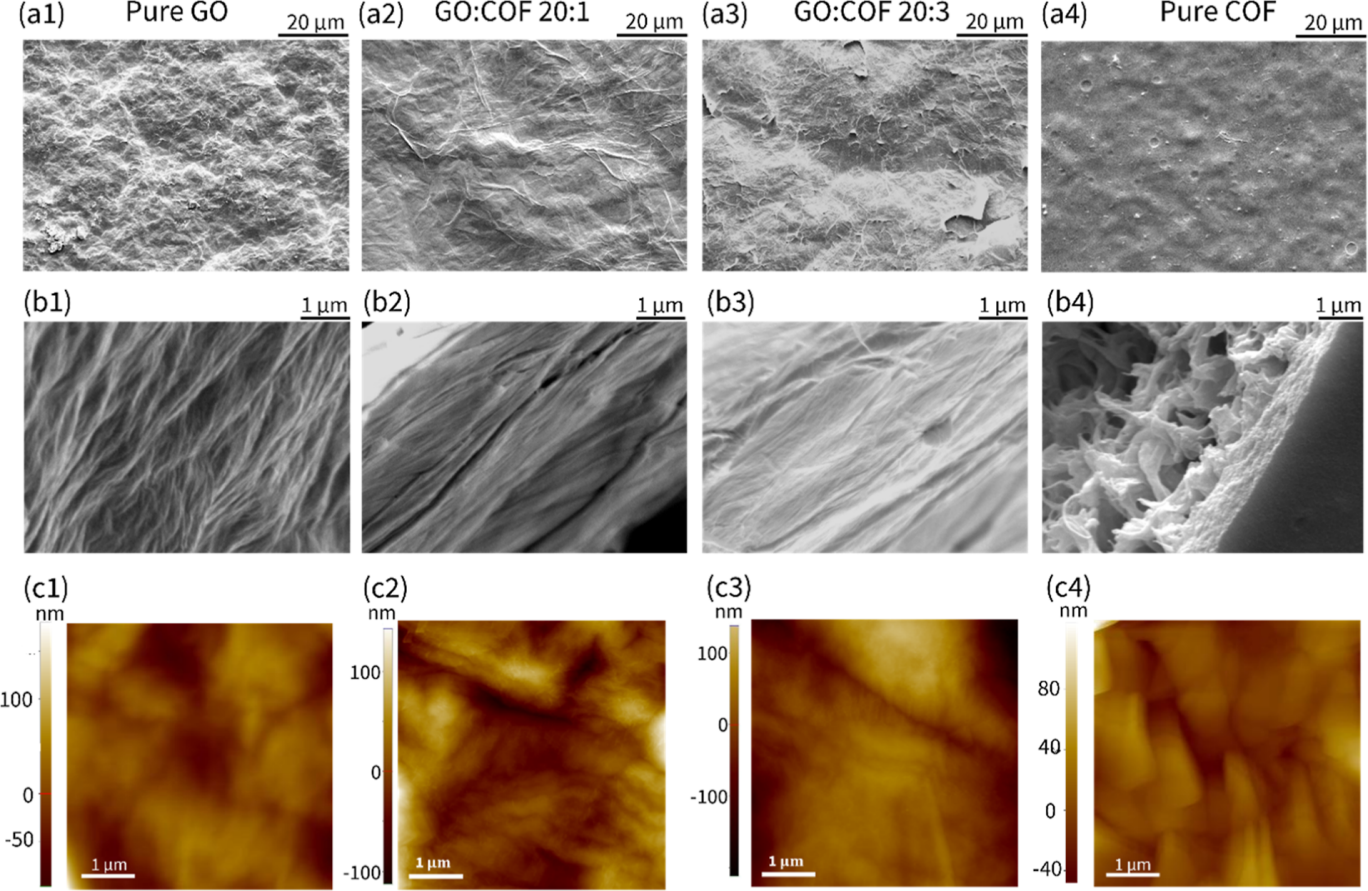
Morphological observations of different membranes. (a) Surface SEM images for (a1) pure GO membrane, (a2) GO/COF = 20:1 membrane, (a3) GO/COF = 20:3 membrane, and (a4) pure COF membrane. (b) Cross-sectional SEM images of (b1) pure GO membrane, (b2) GO/COF = 20:1 membrane, (b3) GO/COF = 20:3 membrane, and (b4) pure COF membrane. Scale bars are shown at the top right corner of the images. (c) AFM images of (c1) pure GO membrane, (c2) GO/COF = 20:1 membrane, (c3) GO/COF = 20:3 membrane, and (c4) pure COF membrane. The average roughness (Ra) scale is listed at the left side of each image.

We further investigated the crystal structure and chemical composition of the as-synthesized GO/COF hybrid membranes. X-ray diffraction (XRD) of the pure COF exhibited characteristic diffraction peaks at 4.7° and 28.0°, corresponding to the (100) and (001) crystallographic planes of the NUS-9 COF, respectively (Figure S1).^65^ The XRD of the GO membrane displayed a prominent peak at 11.4° (Figure 3a), associated with the (001) facet of GO and an interlayer spacing of 0.77 nm.^57^ As the COF content increased in the hybrid membranes, the GO diffraction peak shifted to lower angles (Figure 3a), specifically to 10.7° and 10.2° for GO ratios of 20:1 and 20:3, respectively. Notably, the characteristic COF peaks became less discernible with higher GO content in the hybrid membranes, likely due to the dominant mass fraction of GO relative to the COF. The Fourier transform infrared spectroscopy (FTIR) spectra of the COFs (Figure 3b) exhibited characteristic peaks at 1574 cm^–1^ and 1440 cm^–1^, corresponding to the stretching vibrations of C=C bonds and O=S=O groups, respectively.^65,68^ Similarly, the FTIR spectra of the GO membrane showed prominent peaks at 1553 cm^–1^ and 1730 cm^–1^ (Figure 3b), attributed to aromatic double bonds and carboxyl groups originating from GO.^57^ In the FTIR spectrum of the GO/COF hybrid membrane (Figure 3b), all characteristic peaks from both GO and the COF were present, indicating that the chemical integrity of the hybrid membrane was preserved after vacuum filtration. The X-ray Photoelectron Spectroscopy (XPS) survey scan of all ratios of GO/COF hybrid membranes revealed characteristic peaks around 530 eV, 397 eV, and 284 eV (Figure S2), corresponding to O 1s, N 1s, and C 1s, respectively.^69,70^ The high-resolution S 2p spectrum further confirmed the presence of sulfur in the NUS-9 COF, with a characteristic peak observed at 165 eV (Figure S3). The high-resolution C 1s spectrum of GO/COF (20:1) exhibits peaks at 283.5, 284.6, and 286.0 eV (Figure 3c), corresponding to C–C/C=C, C–O–C, and C=O bonds, respectively.^71^ The peak around 286.0 eV in GO/COF is more prominent than that in the pure GO membrane (Figure 3c), likely due to the contribution of C=O components from the ketoenamine structure in the COF.^72^ The spectrum of O 1s further confirms the presence of the O=C bonds and a small proportion of the O–C bonds (Figure S4).

**Figure 3:**
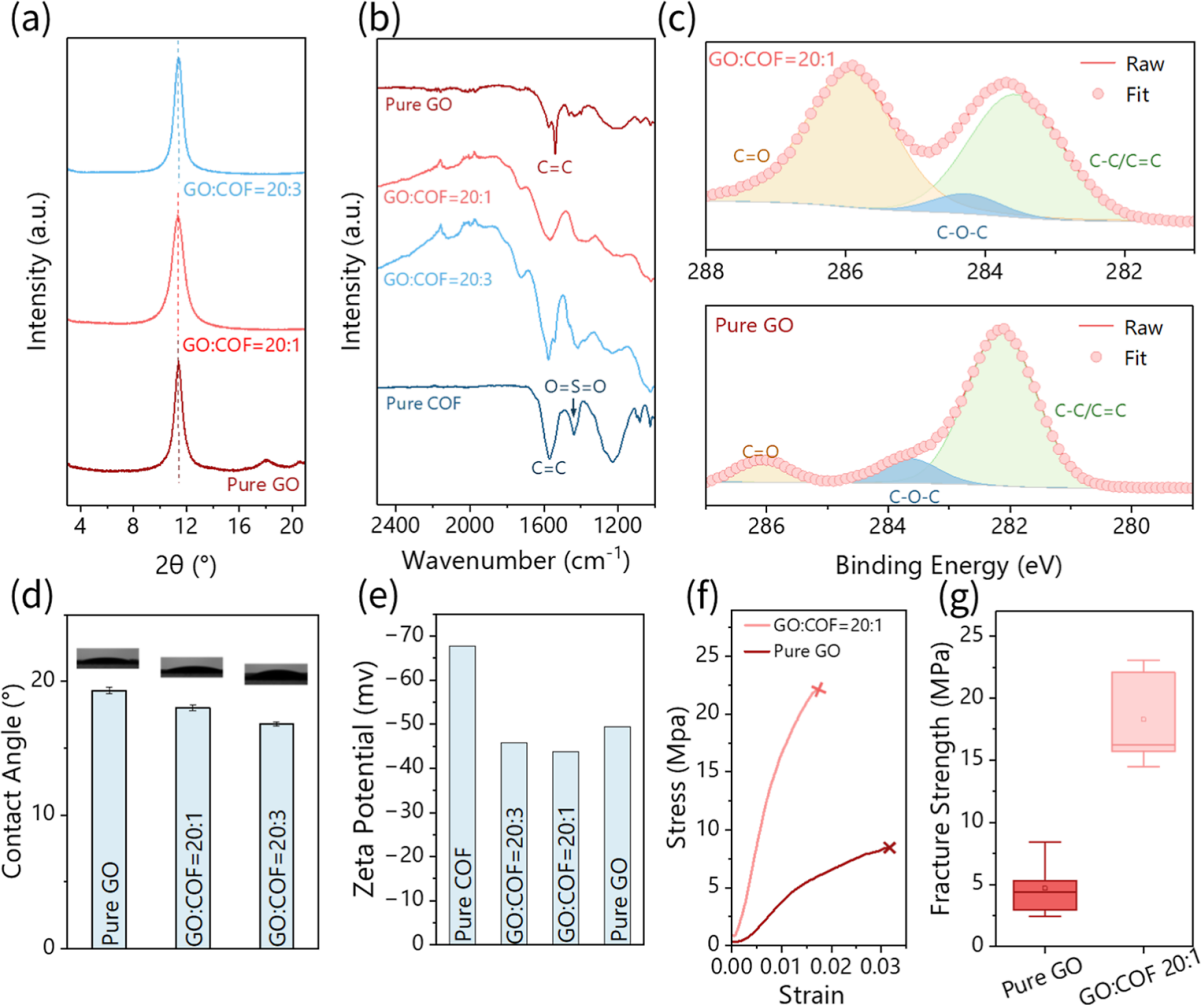
Characterization results of GO/COF and other membranes. (a) XRD patterns of GO/COF = 20:1, CO/COF = 20:3, and pure GO membranes. (b) FT-IR spectra of pure GO, CO/COF = 20:1, CO/COF = 20:3, and pure COF membranes. (c) XPS spectra of C from GO/COF = 20:1 and pure GO membranes. (d) The contact angle of membranes, inserted with water drop images. (e) Zeta potential of different membranes. (f) Tensile stress–strain curve of GO/COF = 20:1 and pure GO membranes. (g) Fracture strength of GO/COF = 20:1 and pure GO membranes.

The surface hydrophilicity of the GO and GO/COF hybrid membranes was further characterized by using contact angle measurements (Figure 3d). The pure GO membrane exhibited a high degree of hydrophilicity, with a small contact angle of 19.3°, attributed to the abundance of hydrophilic carboxyl (–COOH) groups in GO.^57^ As the COF content increased in the hybrid membranes, the contact angle decreased further, reaching 18.0° for a GO-to-COF ratio of 20:1 and 16.8° for a GO-to-COF ratio of 20:3. This enhancement in hydrophilicity with increasing COF content is likely due to the introduction of hydrophilic sulfonic (–SO_3_ H) groups from the COF.^73^

The surface charge density of the membrane is critical for ion selectivity. To evaluate this, we measured the zeta potential of GO, the COF, and their mixture suspensions (Figure 3e). Both pure GO and pure COF nanosheets exhibited negative charges, with zeta potentials of −67.7 and −49.4 mV, respectively. The zeta potentials of the hybrid suspensions, GO/COF 20:1 and GO/COF 20:3, remained negative, measured at −43.8 and −45.8 mV, respectively. The negative charged membrane is advantageous for cation transport, potentially enhancing ion affinity during ED operations.^74^ The negative zeta potential values (−43.8 to −67.7 mV) originate from both the carboxyl groups in GO and the sulfonic acid groups in the COF. While these negative charges enhance overall cation flux through electrostatic attraction, the selectivity between different cations is governed by the hydrated pore size of the COF channels (∼0.7 nm) rather than charge effects, as evidenced by the maintained Na^+^/Ca^2+^ selectivity even in high ionic strength conditions (387 g/L TDS) where charge screening is significant.

To evaluate the mechanical properties of the synthesized GO/COF hybrid membranes, tensile tests were conducted to assess their performance under the applied stress. Figure 3f presents the representative stress–strain curves for the tested membranes. The fracture stress values were determined to be 4.70 ± 2.38 MPa for pure GO and 18.30 ± 3.96 MPa for the GO/COF = 20:1 membranes (Figure 3g). Meanwhile, the pure COF film was not capable of forming a free-standing structure due to its weak mechanical properties. These findings suggest that the incorporation of COF nanosheets between GO nanosheets significantly enhances the fracture strength of the hybrid membranes.

We further evaluated the ion selectivity of the as-synthesized membranes in ED cells, as shown in Figure 1d. A feed solution containing a mixture of 20 mM Na^+^, Li^+^, and Ca^2+^ was used to assess the ion transport behavior. As depicted in Figure 4a, the pure GO membrane exhibited relatively high ion fluxes for all tested cations, with Na^+^, Li^+^, and Ca^2+^ fluxes of 148.10 ^2^, 80.25 ^2^, and 91.24 mmol/h/m^2^, respectively. This can be attributed to the aggregation and swelling of the GO membrane, as observed in SEM and AFM images, which increase the interlayer channel size of the membrane, thereby facilitating ion diffusion and reducing selectivity. In contrast, both GO/COF and pure COF membranes (Figure 4a) demonstrated preferential selectivity for Na^+^ while effectively excluding Ca^2+^. For instance, the GO/COF = 20:1 membrane exhibited a Na^+^ flux of 14.81 mmol/h/m^2^, a Li^+^ flux of 6.69 mmol/h/m^2^, and a significantly reduced Ca^2+^ flux of 0.97 mmol/h/m^2^. This enhanced Na^+^ selectivity in GO/COF membranes is primarily attributed to the size exclusion effect imposed by the intrinsic pore size of the COFs. Specifically, in NUS-9, the sulfonic acid (–SO_3_H) functional groups within the COF pores undergo hydration in aqueous environments, forming hydration shells that further narrow the effective ion transport channels to approximately 0.7 nm.^41^ The sulfonic acid (–SO_3_H) groups in the NUS-9 COF create a hydration-mediated size exclusion mechanism. In aqueous environments, each –SO_3_H group coordinates with 3–4 water molecules, forming stable hydration shells that effectively constrict the COF pore aperture from its crystallographic dimension of ∼1.0 nm to an effective size of ∼0.7 nm.^41,75,76^ This hydration-induced pore narrowing is supported by the enhanced membrane hydrophilicity (contact angle decreasing from 19.3° for pure GO to 16.8° for GO/COF = 1:1) and the sharp discrimination between ions of different hydrated sizes. Given that the hydrated diameters of Na^+^, Li^+^, and Ca^2+^ are 0.56–0.72 nm, 0.73–0.76 nm, and 0.82–0.96 nm, respectively,^41,75,76^ the hydrated COF channels permit Na^+^ transport (flux: 14.81 mmol/h/m^2^) while largely excluding Ca^2+^ (flux: 0.97 mmol/h/m^2^), achieving a 15-fold selectivity difference (Figure S5). Additionally, both the COF and GO/COF membranes exhibited high charge efficiency, with values of 80.4% and 62.8%, respectively (Figure 4b). Notably, the charge efficiency of the pure GO membrane exceeded 100%, further indicating that ion transport primarily occurs by diffusion via defects or cracks formed due to the swelling of the GO membrane. This proposed mechanism is further corroborated by single-ion diffusion tests on the GO/COF = 20:1 membrane in the absence of an applied electric field (Figure 4c). Na^+^ exhibited the highest ion flux of 1.79 mmol/h/m^2^, whereas the Ca^2+^ flux was substantially lower at 0.03 mmol/h/m^2^, confirming the strong size-exclusion effect of the COF-modified membrane. It should be noted that this diffusion test is not intended to simulate ED operational conditions but rather serves as a complementary characterization method to evaluate the membrane’s intrinsic selectivity under simplified conditions without electric field interference. The consistent selectivity observed under both electrodriven and concentration-driven conditions confirms that our membrane’s monovalent/divalent discrimination originates from the stable size-exclusion mechanism of COF nanochannels rather than field-dependent phenomena, validating the robustness of our membrane design.

**Figure 4:**
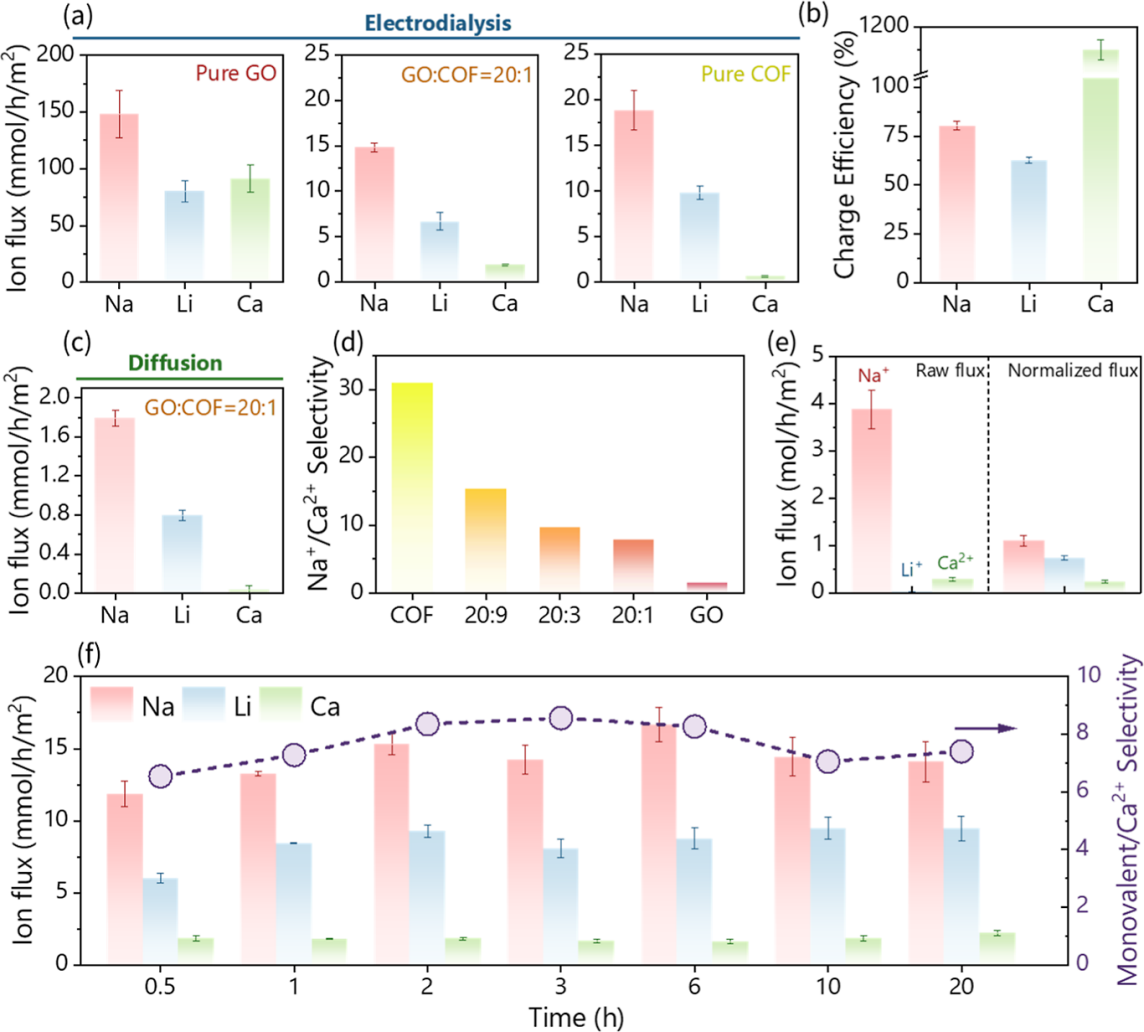
Ion permeation tests. (a) Ion flux of Na^+^, Li^+^, and Ca^2+^ measured from pure GO, GO/COF = 20:1, and pure COF membranes under the ED scenario. (b) Charge efficiencies of different membranes. (c) Ion flux of Na^+^, Li^+^, and Ca^2+^ measured from the GO/COF = 20:1 membrane in the diffusion test. (d) Na^+^/Ca^2+^ selectivity comparisons among membranes. (e) The raw and normalized ion flux of Na^+^, Li^+^, and Ca^2+^ measured from the GO/COF = 20:1 membrane using synthetic brine as feed solution. (f) Ion flux of Na^+^, Li^+^, and Ca^2+^ from 20 h of extended ED experiments with monovalent/Ca^2+^ selectivity.

Moreover, the ion separation performance is strongly influenced by the GO-to-COF ratio. As shown in Figure 4d, increasing the GO/COF ratio from 20:1 to 20:9 leads to a substantial increase in the measured Na^+^/Ca^2+^ selectivity, rising from 7.83 to 15.34. These results demonstrate that the composition of the GO/COF composite membrane plays a critical role in governing ion transport behavior, underscoring its promise for selective ion separation applications in electrodialysis.

To demonstrate the stability and practical applicability of the GO/COF composite membrane, we evaluated a membrane with a GO/COF ratio of 20:1 using a hypersaline synthetic brine, mimicking the major cation composition of actual Salton Sea geothermal brine,^77^ with an ultrahigh total dissolved solids (TDS) content of 386,700 mg/L (Figure 4e). Na^+^ exhibited the highest ion flux of 3879 mmol/h^1^/m^2^, dominating the overall ion transport. In contrast, Li^+^ and Ca^2+^ demonstrated substantially lower fluxes of 36.82 and 291.5 mmol/h^1^/m^2^, respectively. Since these flux differences partially reflect the varying initial concentrations of each cation, we calculated concentration-normalized fluxes to enable a more rigorous comparison (Figure 4e). After normalization, the selectivity ratios of Na^+^/Ca^2+^ (7.84) and Li^+^/Ca^2+^ (5.28) remained consistent with previous measurements, confirming stable membrane performance and maintained selectivity even under hypersaline conditions. To further assess the long-term stability of the GO/COF membrane, we conducted extended testing of the same membrane over 20 h, ten times longer than standard ion permeation experiments (Figure 4f). Throughout the testing period, all cation fluxes remained stable across multiple sampling points, with selectivity values maintaining consistency, further validating the membrane’s durability for practical applications.

GO/COF membrane performance was compared with that of state-of-the-art membranes reported in the literature (Table S1). While some modified membranes achieve higher absolute selectivity, GO/COF membranes demonstrate easily variable selectivity from 7.88 to 15.34 simply by adjusting the GO/COF ratio, unlike conventional membranes, which require complex modifications or different materials to alter selectivity. In addition, the low ion flux exhibited in this study was due to the low current density of 20 μA/cm^2^ applied, which was well below industrial levels of 10–100 mA/cm^2^. Higher flux could be achieved with a more intense electrical field given >75% current efficiency, indicating strong potential for high-flux operation when scaled to industrial conditions where ion flux scales proportionally with current density. Combined with superior mechanical properties (18.30 ± 3.96 MPa) and demonstrated stability under hypersaline conditions, our GO/COF membranes represent a promising platform for versatile electrodialysis applications where operational flexibility and scalability are of paramount importance.

## Conclusion

In conclusion, we successfully demonstrated a robust electrodialysis membrane by stacking GO nanosheets and sulfonate-functionalized COF nanosheets. The membrane exhibited excellent monovalent ion selectivity and efficient ion transport, with a nacre-inspired layered structure providing an enhanced mechanical strength. Material characterization using SEM, AFM, XRD, and FTIR confirmed the formation of a compact, well-aligned layered structure of GO/COF nanosheets, which is critical for enhanced ion separation. Mechanical testing showed that incorporating COF nanosheets significantly enhanced the membrane’s structural robustness, increasing fracture strength from 4.70 ± 2.38 MPa for pure GO to 18.30 ± 3.96 MPa for the GO/COF = 20:1 membrane. Pure COF films could not form free-standing structures due to their weak mechanical properties, highlighting the mechanical reinforcement achieved by GO–COF integration. The GO/COF composite membrane achieved Na^+^/Ca^2+^ and Li^+^/Ca^2+^ selectivity ratios of 15.34 and 6.99, respectively. By adjustment of the relative composition of the COF and GO, monovalent ion selectivity was changed from 7.88 to 15.34 while maintaining a charge efficiency exceeding 75%. These results underscore the potential of GO/COF composite membranes for energy-efficient electrodialysis. The combination of high selectivity and mechanical durability makes this membrane a strong candidate for sustainable water treatment solutions. Future work will focus on scaling up fabrication and evaluating long-term stability under various operational conditions.

## Material and Methods

### Materials

The 0.22 μm nylon filtration membrane was purchased from Millipore. 1,3,5-Triformylphloroglucinol (TFP, ≥97.0%) was purchased from Ambeed. Graphene oxide (GO, 2 mg/mL) was purchased from Tanfeng Tech. Inc., China. Methylene chloride (CH_2_Cl_2,_ 99.8%), acetic acid (HOAc, ≥99%), 2,5-diaminobenzenesulfonic acid (DABA, ≥97.0%), and *N*,*N*-dimethylformamide (DMF, anhydrous, 99.8%) were purchased from Sigma-Aldrich. Sodium chloride (NaCl, ≥99%), calcium chloride (CaCl_2_, 93%), and lithium chloride (LiCl, 99%) were purchased from Thermo Scientific. The electrodialysis flow cell was made in our lab. All the chemicals were used as received without further purification unless specific treatment was mentioned.

### Synthesis of NUS-9 COF Nanosheets

The synthesis of NUS-9 COF nanosheets (Figure 1a) via the liquid–liquid interfacial polymerization method followed the reported research.^65^ Solution A: 21 mg of TFP was dissolved in 50 mL of CH_2_Cl_2_. Solution B: 7.2 g of HOAc was dissolved in 50 mL of DI water. Solution C: 28 mg of DABA was dissolved in 50 mL of DMF. Solution A was added to a 150 mL beaker, solution B was added on top of solution A dropwise. Then, solution C was added on top of solution B drop by drop. The reaction was performed at room temperature for 7 days. The second bottom layer of aqueous solution was then placed on a semipermeable membrane, which was then immersed in a 1 L beaker containing DI water. A purified aqueous dispersion of COF nanosheets was obtained after changing the DI water several times until the pH of the solution reached 6.

### GO/COF Membrane Fabrication

After determination of COF concentration suspended in DI water, a certain volume of 0.3 g L^–1^ COF suspension was mixed with 2 g L^–1^ GO suspension and sonicated 30 min for complete mixing. Both the COF and GO suspension volumes for mixing followed the designed ratio (20:1, 20:3, 20:9) to reach a total volume of 12 mL. The mixture was then filtered assisted by vacuum (10^–3^ Torr) onto a 0.22 μm nylon filtration membrane to form a GO/COF membrane (Figure 1b). After filtration, the as-synthesized GO/COF membranes were soaked in DI water for substrate detachment and preserved in water before use (Figure 1c). The pure COF membrane couldn't be made free-standing, and attempting to detach it from the underlying nylon substrate results in membrane fracture.

### Characterization of Membranes

Surface morphology and cross-section of membranes were investigated by scanning electron microscopy (SEM) using a Helios 660 SEM/FIB (Thermo Fisher Scientific, USA). The X-ray diffraction (XRD) pattern of membranes was obtained using a Rigaku D/Max Ultima II Powder X-ray diffractometer with Cu Kα radiation, operating at 40 kV and 40 mA and recording from 0° to 20° (in 2θ). The chemical composition bonding conditions of the membrane were determined by X-ray Photoelectron Spectroscopy (XPS, PHI Quentera, ULVAC-PHI, Japan), recorded from 50 to 650 eV binding energy, and Fourier-Transform Infrared Spectroscopy (FTIR, Nicolet, Thermo Fisher Scientific), recorded from 400 to 4000 cm^–1^ wavenumber. Zeta potential was measured using the SurPASS electrokinetic analyzer (Anton Paar, Graz, Austria) equipped with an adjustable gap cell. The surface’s contact angle measurements were obtained using a Ramé–Hart Model 200 imaging system. Tensile tests of the GO/COF thin film samples were conducted using an in-SEM Deben MICROTEST stage equipped with a 200 N load cell at an extension rate of 0.5 mm/min. Each sample was cut using a 3 cm long and 5 mm wide dog-bone-shaped die according to the ASTM standard D412. The thickness of the films was measured by using SEM cross-sectional images. Three samples were tested for each mechanical measurement, and error bars were calculated based on the standard deviation from triplicate measurements. These experimental values were input into each respective load-displacement plot, which was analyzed using a custom MATLAB code. The fracture strength and fracture strain were recorded.

### Ion Separation Test

The ion separation test was conducted in a customized ED cell with a 5 cm^2^ effective membrane area. A 3-chamber ED cell (Figure 1d) was utilized, and the GO/COF membrane was placed between the right and middle chambers, while a commercial AEM was placed between the middle and left chambers. A premade feed solution containing 20 mM NaCl, 20 mM LiCl, and 20 mM CaCl_2_ was pumped into the middle chamber, while the right chamber was added with DI water and regarded as an extractant. The left chamber was added with the anolyte (NaCl) for conductivity purposes. 20 μA/cm^2^ current density was applied during the ED test. Feed and extractant samples were taken at time intervals. The diffusion test was conducted for consistency without applying an electrical field.

## Supplementary Material



## Notes

Detailed information on PXRD characterization of the COF sheet (Figure S1); XPS survey, S 2s, and O 1s scan of pure GO, the COF sheet, and the GO/COF membrane (Figure S2–S4); schematic of the proposed separation mechanism of the GO/COF membrane (Figure S5); and membrane performance comparison (Table S1) (PDF)
