# Supplementary material for: Mechanically Strengthened Graphene Oxide: Covalent Organic Framework Membranes for Monovalent/Divalent Cation Selectivity via Electrodialysis
Source: ACS Nano. 2026 Jan 21;20(4):3632–41. doi: 10.1021/acsnano.5c17289 (PMC12875026; doi:10.1021/acsnano.5c17289)
Supplement: Supplementary file 1 [file nn5c17289_si_001.pdf]

Supporting Information

Mechanically Strengthened Graphene Oxide:Covalent Organic Framework Membranes for Monovalent/Divalent Cation Selectivity via Electrodialysis

Yuren Feng[1,2†], Xiaoyin Tian[1,3], [†] Yifan Zhu[1,3*†], Qiyi Fang[3], Rodolfo Cantu[3], Bongki Shin[3], Xiaochuan Huang[1,2], Xintong Weng[3], Xiang Zhang[3], Yunhao Zhang[2], Doug Steinbach[3], Qing Ai[3], Tsai-Hsuan Chen[2,4], Yimo Han[3], Pulickel M Ajayan[1,3,5,6], Qilin Li[1,2,3,5,6,7*], Jun Lou[1,3,6,7,8*]

1 NSF Nanosystems Engineering Research Center Nanotechnology-Enabled Water Treatment, Rice University, 6100 Main Street, Houston 77005, United States

2 Department of Civil and Environmental Engineering, Rice University, 6100 Main Street, Houston 77005, United States

3 Department of Materials Science and NanoEngineering, Rice University, 6100 Main Street, Houston, TX 77005, United States

4 Graduate Institute of Environmental Engineering, National Taiwan University, No. 1, Sec. 4. Roosevelt Rd., Taipei 10617, Taiwan

5 Department of Chemical & Biomolecular Engineering, Rice University, 6100 Main Street, Houston, 77005, United States

6 Rice Advanced Materials Institute, Rice University, 6100 Main Street, Houston, Texas 77005, United States

7 Rice WaTER Institute, Rice University, 6100 Main Street, Houston, TX 77005, United States

8 Department of Chemistry, Rice University, 6100 Main Street, Houston, Texas 77005, United States

†These authors contributed equally to this work

Corresponding authors:

Yifan Zhu: yz130@rice.edu Qilin Li: qilin.li@rice.edu Jun Lou: Jun.lou@rice.edu

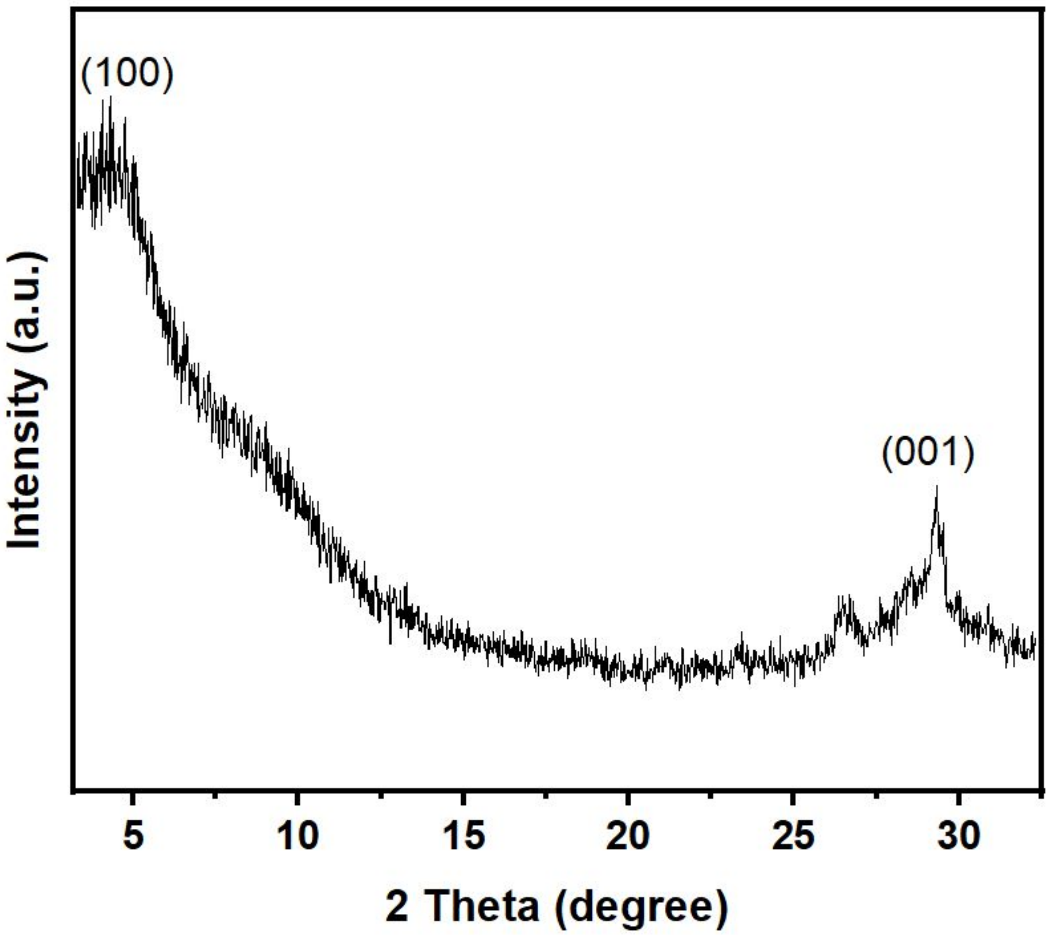

Figure S1. PXRD of COF nanosheets.

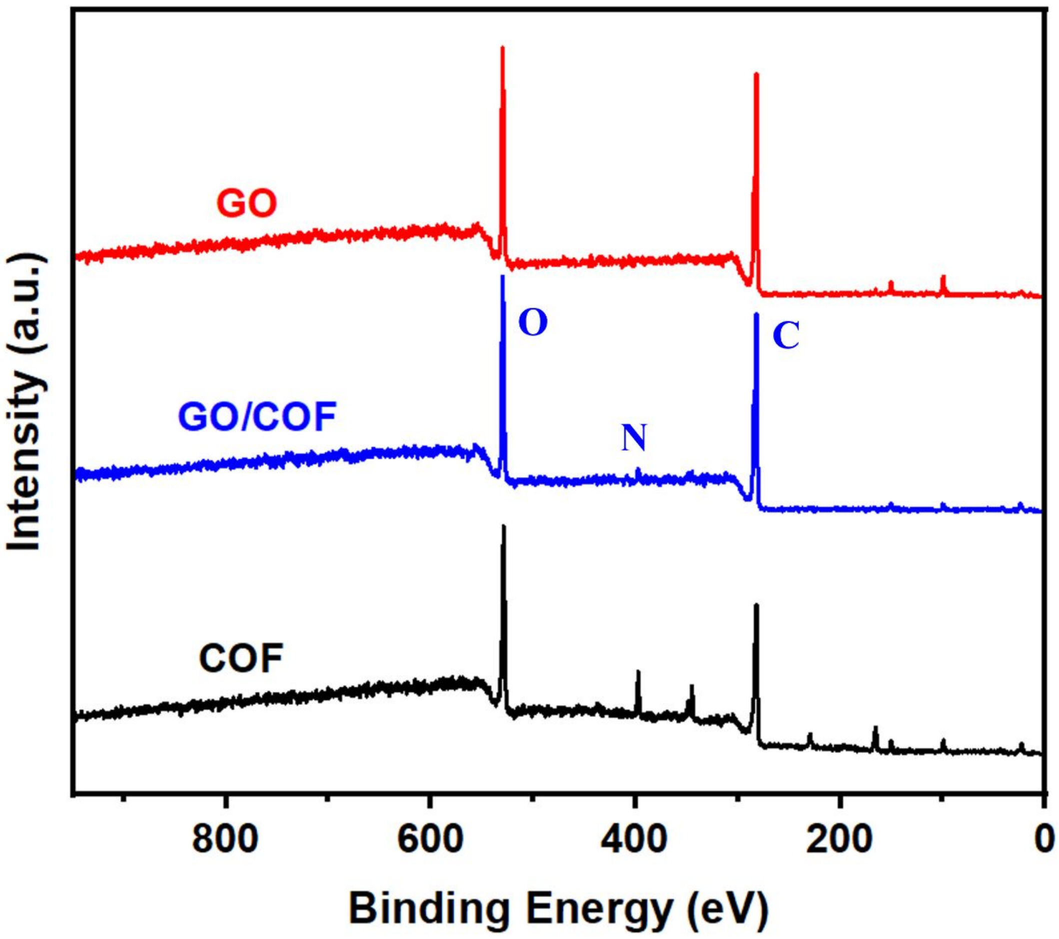

Figure S2. XPS survey scan of GO, GO-COF (20:1) and COF.

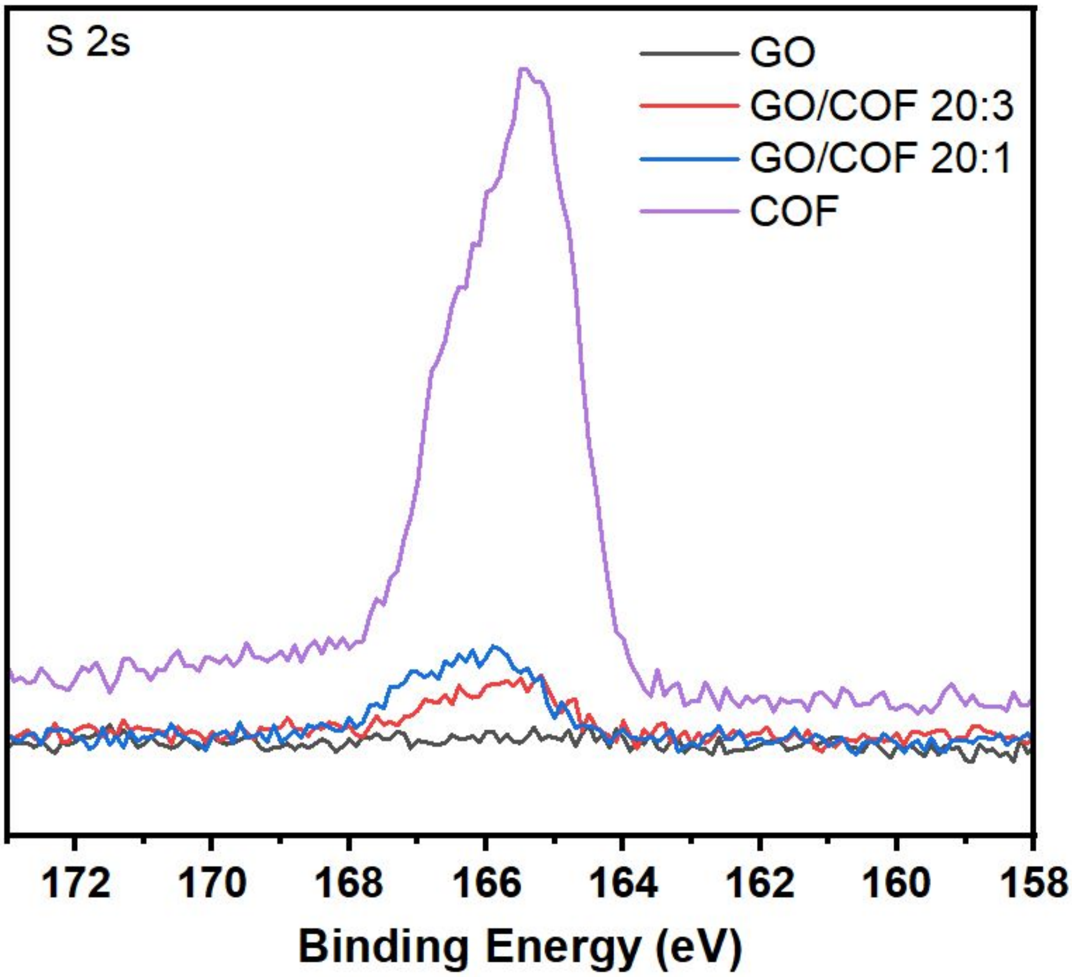

Figure S3. High resolution XPS S2s scan of GO, GO-COF and COF.

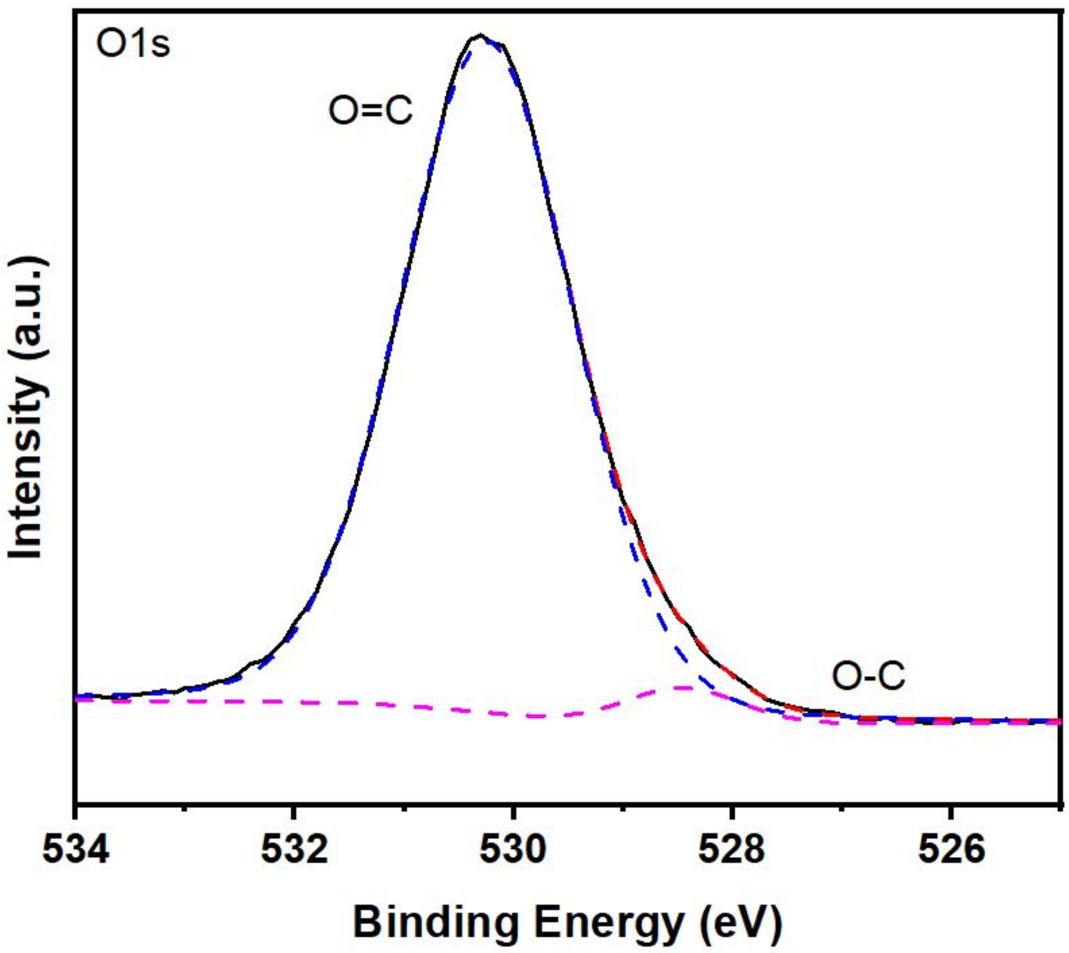

Figure S4. High resolution XPS O1S scan of GO-COF = 20:1.

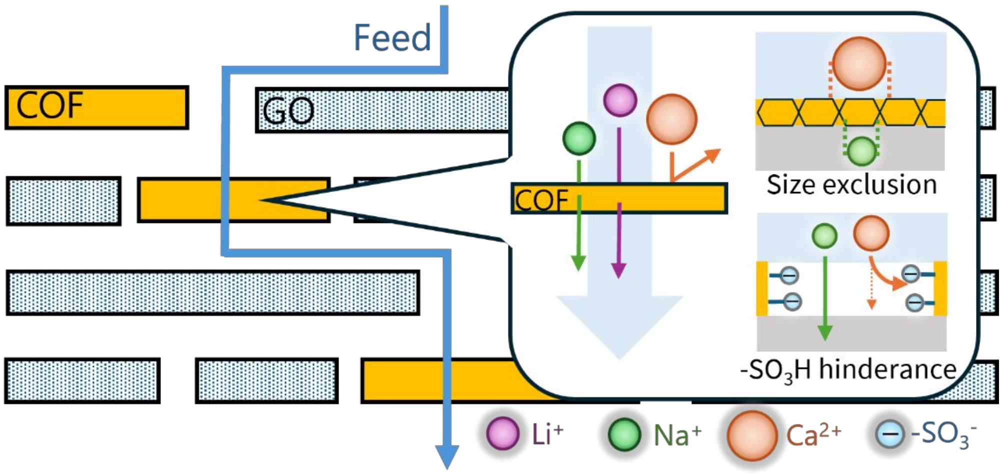

Figure S5. Schematic illustration of the proposed membrane selectivity mechanism.

Table S1. The membrane performance comparison.

Membrane	Monovalent conc. (mM)	Mono/divalent molar ratio	Selectivity	Ref.
**GO-COF**	20	0.04	7.8-15.3	This work
**MCPMs**	50	1	1.4	Ref [1]
**MXene**	200	1	8.8	Ref [2]
**CIMS**	20	0.01-0.02	8.6	Ref [3]
**CSO**	820	0.16	18.9	Ref [4]
**CIMS**	50	0.03-1	7.17	Ref [5]
**ZIF-8-PDA**	10	1	0.41	Ref [6]
**TFSI**	320	0.065	249.7	Ref [7]
**PIM**	0.29	0.01-1	0.9	Ref [8]
**VCT**	0.1	-	58.9	Ref [9]
**EDNF**	459	8.8	7	Ref[10]
**MCPM**	100	1	7.4	Ref[11]
**QPO/DAN-SA**	100	1	58.4	Ref[12]
**SPPO CEM**	100	1	12.7	Ref[13]
**SPSF CEM**	50	1	6.0	Ref[14]

Reference

(1) Pang, X.; Yu, X.; He, Y.; Dong, S.; Zhao, X.; Pan, J.; Zhang, R.; Liu, L. Preparation of Monovalent Cation Perm-Selective Membranes by Controlling Surface Hydration Energy Barrier. *Separation and Purification Technology* **2021**, *270*, 118768. https://doi.org/10.1016/j.seppur.2021.118768.

(2) Ren, C. E.; Hatzell, K. B.; Alhabeb, M.; Ling, Z.; Mahmoud, K. A.; Gogotsi, Y. Charge- and Size-Selective Ion Sieving Through Ti3C2Tx MXene Membranes. *J. Phys. Chem. Lett.* **2015**, *6* (20), 4026–4031. https://doi.org/10.1021/acs.jpclett.5b01895.

(3) Ji, Z.; Chen, Q.; Yuan, J.; Liu, J.; Zhao, Y.; Feng, W. Preliminary Study on Recovering Lithium from High Mg2+/Li+ Ratio Brines by Electrodialysis. *Separation and Purification Technology* **2017**, *172*, 168–177. https://doi.org/10.1016/j.seppur.2016.08.006.

(4) Nie, X.-Y.; Sun, S.-Y.; Song, X.; Yu, J.-G. Further Investigation into Lithium Recovery from Salt Lake Brines with Different Feed Characteristics by Electrodialysis. *Journal of Membrane Science* **2017**, *530*, 185–191. https://doi.org/10.1016/j.memsci.2017.02.020.

(5) Chen, Q.-B.; Ji, Z.-Y.; Liu, J.; Zhao, Y.-Y.; Wang, S.-Z.; Yuan, J.-S. Development of Recovering Lithium from Brines by Selective-Electrodialysis: Effect of Coexisting Cations on the Migration of Lithium. *Journal of Membrane Science* **2018**, *548*, 408–420. https://doi.org/10.1016/j.memsci.2017.11.040.

(6) Yu, H.; Hossain, S. M.; Wang, C.; Choo, Y.; Naidu, G.; Han, D. S.; Shon, H. K. Selective Lithium Extraction from Diluted Binary Solutions Using Metal-Organic Frameworks (MOF)-Based Membrane Capacitive Deionization (MCDI). *Desalination* **2023**, *556*, 116569. https://doi.org/10.1016/j.desal.2023.116569.

(7) Liu, G.; Zhao, Z.; He, L. Highly Selective Lithium Recovery from High Mg/Li Ratio Brines. *Desalination* **2020**, *474*, 114185. https://doi.org/10.1016/j.desal.2019.114185.

(8) Paredes, C.; Rodríguez de San Miguel, E. Selective Lithium Extraction and Concentration from Diluted Alkaline Aqueous Media by a Polymer Inclusion Membrane and Application to Seawater. *Desalination* **2020**, *487*, 114500. https://doi.org/10.1016/j.desal.2020.114500.

(9) Razmjou, A.; Eshaghi, G.; Orooji, Y.; Hosseini, E.; Korayem, A. H.; Mohagheghian, F.; Boroumand, Y.; Noorbakhsh, A.; Asadnia, M.; Chen, V. Lithium Ion-Selective Membrane with 2D Subnanometer Channels. *Water Research* **2019**, *159*, 313–323. https://doi.org/10.1016/j.watres.2019.05.018.

(10) Ge, L.; Wu, B.; Li, Q.; Wang, Y.; Yu, D.; Wu, L.; Pan, J.; Miao, J.; Xu, T. Electrodialysis with Nanofiltration Membrane (EDNF) for High-Efficiency Cations Fractionation. *Journal of Membrane Science* **2016**, *498*, 192–200. https://doi.org/10.1016/j.memsci.2015.10.001.

(11) He, Y.; Ge, L.; Ge, Z.; Zhao, Z.; Sheng, F.; Liu, X.; Ge, X.; Yang, Z.; Fu, R.; Liu, Z.; Wu, L.; Xu, T. Monovalent Cations Permselective Membranes with Zwitterionic Side Chains. *Journal of Membrane Science* **2018**, *563*, 320–325. https://doi.org/10.1016/j.memsci.2018.05.068.

(12) Yang, S.; Liu, Y.; Liao, J.; Liu, H.; Jiang, Y.; Van der Bruggen, B.; Shen, J.; Gao, C. Codeposition Modification of Cation Exchange Membranes with Dopamine and Crown Ether To Achieve High K+ Electrodialysis Selectivity. *ACS Appl. Mater. Interfaces* **2019**, *11* (19), 17730–17741. https://doi.org/10.1021/acsami.8b21031.

(13) Afsar, N. U.; Ji, W.; Wu, B.; Shehzad, M. A.; Ge, L.; Xu, T. SPPO-Based Cation Exchange Membranes with a Positively Charged Layer for Cation Fractionation. *Desalination* **2019**, *472*, 114145. https://doi.org/10.1016/j.desal.2019.114145.

(14) Irfan, M.; Xu, T.; Ge, L.; Wang, Y.; Xu, T. Zwitterion Structure Membrane Provides High Monovalent/Divalent Cation Electrodialysis Selectivity: Investigating the Effect of Functional Groups and Operating Parameters. *Journal of Membrane Science* **2019**, *588*, 117211. https://doi.org/10.1016/j.memsci.2019.117211.
